# Supplementary material for: WordCommentsAnalyzer: A windows software tool for qualitative research
Source: F1000Res. 2018 Sep 4;7:536. Originally published 2018 May 3. [Version 2] doi: 10.12688/f1000research.14819.2 (PMC6137411; doi:10.12688/f1000research.14819.2)
Supplement: Supplementary file 3 [file f1000research-7-17614-s0002.tgz › b2a79496-4ada-47c4-9682-a99ce867836d.zip!/Eight Iranian Health Journals/Journal of Education and Community Health_10.docx]

© Journal of Education and Community Health. This work is licensed under CC BY-NC 4.0 (https://creativecommons.org/licenses/by-nc/4.0/).

Volume & Issue: دوره 2 - شماره 3

Title: تجارب دانش آموزان دبیرستانی در مورد پیش بینی کننده های مصرف دخانیات : یک تحلیل محتوی کیفی نظریه محور

http://jech.umsha.ac.ir/article-1-129-fa.html

سابقه و هدف: مصرف دخانیات به عنوان یکی از عوامل خطر ساز مهم و افزایش دهنده بار کلی بیماری ها در دنیا به شمار می رود. با توجه به روند رو به افزایش مصرف دخانیات، پژوهش حاضر با هدف تبیین تجارب دانش آموزان دبیرستانی از تعیین کننده های مصرف و عدم مصرف دخانیات (سیگار و قلیان) بر اساس تئوری انگیزش محافظت انجام شد. مواد و روش ها: مطالعه حاضر یک پژوهش کیفی از نوع تحلیل محتوا می باشد که به مدت پنج ماه از اول آذر ماه تا پایان فروردین ماه سال1394 در دبیرستان های پسرانه شهرستان نوشهر انجام شد. داده ها به صورت مصاحبه های عمیق نیمه ساختارمند و فردی از 21 دانش آموزان پسر دبیرستانی شامل 7 نفر مصرف کننده سیگار، 7 نفر مصرف کننده قلیان و 7 نفر بدون مصرف هرگونه ماده دخانی به دست آمد. آنالیز داده ها با استفاده از روش آنالیز محتوا کیفی هدایت شده (تئوری محور) صورت گرفت. یافته‌ها: تحلیل داده ها منجر به استخراج تعداد 99 کد اولیه گردید که در 9 طبقه از پیش تعیین شده تئوری انگیزش محافظت شامل (حساسیت درک شده، شدت درک شده، ترس، خودکارآمدی درک شده، هزینه پاسخ، کارآمدی پاسخ درک شده، پاداش درک شده بیرونی، پاداش درک شده درونی، انگیزش محافظت) قرار گرفتند. یافته های پژوهش حاضر نشان داد که مهمترین پیش بینی کننده‌های مصرف دخانیات سازه های هزینه پاسخ و پاداش های درک شده بالا و مهمترین پیش بینی کننده های عدم مصرف دخانیات حساسیت درک شده و شدت درک شده و خودکارآمدی بالای دانش آموزان بودند. نتیجه‌گیری: یافته های پژوهش نشان داد فشار همسالان، حضور در جمع دوستان مصرف کننده دخانیات و نبود تفریحات جایگزین از مهمترین عوامل مصرف دخانیات در بین دانش آموزان می باشند. بنابراین پیشنهاد می گردد برنامه ریزان حوزه سلامت، مداخلات جامعی را به منظور بهبود عوامل فردی و محیطی موثر در مصرف دخانیات طراحی و اجرا نمایند تا بتوانند مصرف سیگار و قلیان را  کاهش دهند.

Title: اثربخشی بازی درمانی گروهی بر علائم لجبازی- نافرمانی کودکان

http://jech.umsha.ac.ir/article-1-141-fa.html

سابقه و هدف: با توجه به شیوع اختلال لجبازی-نافرمانی در کودکان و تبدیل شدن آن به اختلال­ های دیگر در صورت برطرف نشدن آن؛‌ هدف پژوهش حاضر بررسی اثربخشی بازی درمانی به روش گروهی بر علائم اختلال لجبازی- نافرمانی کودکان بود. مواد و روش­ ها: این مطالعه مداخله ­ای و از نوع نیمه تجربی بود. در این پژوهش با روش نمونه­ گیری خوشه ­ای 30 نفر انتخاب و به شیوه تصادفی در دو گروه آزمون و کنترل قرار گرفتند. ابزار اندازه­گیری شامل سیاهه رفتاری کودک (CBCL)، ماتریس­های پیشرونده ریون کودک، فرم گزارش معلم (TRF) و یک مصاحبه بالینی با والدین بود. آزمودنی­ها طی 8 جلسه 60 دقیقه­ای که به صورت هفتگی برگزار می­شد، در معرض مداخلات بازی درمانی قرار گرفتند. آزمودنی­ها در سه مرحله پیش از آغاز مداخله، پس از اتمام مداخله و با فاصله دو ماه از اتمام جلسات به عنوان پیگیری مورد ارزیابی قرار گرفتند. برای تحلیل داده ­ها از نرم افزار SPSS18 و روش تحلیل کوواریانس چندمتغیری استفاده شد. یافته­ ها: نتایج تحلیل مانکوا نشان داد که علائم اختلال لجبازی- نافرمانی به گزارش والد و به گزارش معلم در گروه­ آزمون بازی درمانی گروهی در مقایسه با گروه کنترل در مرحله پس­ آزمون کاهش معنی‌داری داشت (P<0.001). همچنین بعد از دو ماه پیگیری نتایج هم چنان پابرجا بود. نتیجه ­گیری: این یافته­ها اثربخشی بازی درمانی گروهی را برای کاهش علائم کودکان دارای اختلال لجبازی- نافرمانی نشان می‌دهد و بر این اساس استفاده از این روش درمانی در مورد این کودکان به درمانگران توصیه می‌شود.

Title: اثربخشی مداخله آموزشی مبتنی بر روش همسان بر میزان آگاهی، نگرش و قصد رفتاری مرتبط با ایدز در دانش آموزان دختر دبیرستان های منطقه 14 شهر تهران

http://jech.umsha.ac.ir/article-1-128-fa.html

سابقه و هدف: بیماری ایدز، یکی از مشکلات بهداشتی در جهان به ویژه در کشورهای  در حال توسعه  می باشد. این مطالعه با هدف بررسی اثربخشی مداخله آموزشی مبتنی بر روش همسان بر میزان آگاهی، نگرش و قصد رفتاری مرتبط با ایدز در دانش آموزان دختر دبیرستان های منطقه 14 شهر تهران انجام شده است. مواد و روش ها: در این مطالعه نیمه تجربی 330 نفر از دانش آموزان دختر به صورت تصادفی چندمرحله ای از بین 18 مدرسه انتخاب شدند. برای جمع آوری اطلاعات از پرسشنامه دارای روایی و پایایی قابل قبول استفاده گردید. قبل از اجرای مداخله از دو گروه کنترل و مداخله پیش آزمون گرفته شد و اطلاعات لازم در باره آگاهی، نگرش  و قصدرفتاری نسبت به رفتارهای پیشگیری کننده ایدز برای طراحی و اجرای مداخله بدست آمد. سپس برنامه آموزشی اجرا و پس از 3 ماه پرسشنامه ها جهت سنجش تاثیر مداخله مجددا تکمیل شدند. برای تجزیه و تحلیل داده ها از نرم افزار آماری -21SPSS و آزمون های آماری تی‌مستقل، تی‌زوجی، کای اسکوئر، همبستگی پیرسون و تحلیل رگرسیون خطی استفاده شد. یافته‌ها: پس از آموزش تغییرات معنی‌داری در میانگین نمرات آگاهی، نگرش و قصد رفتاری گروه مداخله مشاهده شد (0.001>P). سن با نگرش و قصد رفتاری با آگاهی و نگرش همبستگی مستقیم معنی‌داری داشت. آگاهی و نگرش قادر به پیش بینی 41 درصد تغییرات قصد رفتاری در دانش آموزان بودند. نتیجه گیری: یافته های مطالعه حاضر نشان داد که استفاده از روش همسان در مداخلات آموزشی می تواند در افزایش آگاهی و بهبود نگرش و قصد رفتاری نسبت به رفتارهای پیشگیری کننده از ایدز، موثر باشد.

Title: باورهای موثر بر رفتارهای تغذیه ای زنان باردار تحت پوشش مراکز بهداشتی درمانی شهر ساوه

http://jech.umsha.ac.ir/article-1-152-fa.html

سابقه و هدف: اگر چه رژیم غذایی سالم و متعادل در سراسر زندگی مهم است اما در دوران بارداری برای سلامت مادر و جنین از اهمیت بیشتری برخوردار است. مطالعه حاضر با هدف تعیین باورهای موثر بر رفتارهای تغذیه ای زنان باردار تحت پوشش دانشکده علوم پزشکی ساوه انجام شده است.   مواد و روش‌ها: مطالعه حاضر یک مطالعه توصیفی تحلیلی بوده که در آن 260 نفر از زنان باردار  به روش نمونه گیری چند مرحله ای از مراکز بهداشتی درمانی وابسته به دانشکده علوم پزشکی ساوه انتخاب و وارد مطالعه شدند. در پژوهش حاضر روش گردآوری اطلاعات پرسشنامه تنظیم شده بر اساس مدل اعتقاد بهداشتی (حساسیت، شدت، منافع و موانع درک شده، خود کارآمدی و راهنمای عمل) و چک لیست عملکرد در خصوص تغذیه صحیح در دوران بارداری بود. داده ها با استفاده از مصاحبه سازمان یافته جمع آوری و در نهایت با استفاده از نرم افزار 21SPSS و با کمک آزمون های آماری نظیر تی مستقل و آنالیز واریانس یکطرفه تجزیه تحلیل شدند.    یافته‌ها: میانگین سنی شرکت کنندگان 5±27.4 و میانگین سن حاملگی2.4± 5.5 بود. بین منافع و موانع درک شده، حساسیت و شدت درک شده، و خودکارآمدی درک شده با رفتارهای تغذیه ای زنان باردار همبستگی معنی داری ملاحظه شد (0.05>P). به علاوه بین متغیر های سطح سواد، وضعیت اشتغال و تعداد بارداری بارفتارهای تغذیه ای اختلاف معنی داری مشاهده شد (0.05>P). نتیجه گیری: با توجه به اینکه میانگین نمره سازه های مدل اعتقاد بهداشتی در زنان باردار در حد متوسط بود، بنابراین برای بهبود سلامت مادر و کودک، یک برنامه آموزشی دقیق بر اساس مدل اعتقاد بهداشتی برای بالا بردن حساسیت و شدت درک شده و همچنین افزایش خود کارآمدی و منافع درک شده به همراه از بین بردن موانع درک شده پیشنهاد می گردد.

Title: بررسی اثر بخشی آموزش تحلیل رفتار متقابل بر هوش معنوی زوجین شهر اصفهان

http://jech.umsha.ac.ir/article-1-143-fa.html

سابقه و هدف: یکی از انواع هوش‌ها که در سال‌های اخیر مورد توجه قرار گرفته، هوش معنوی است که موجب می‌شود فرد در برابر رویدادها و حوادث زندگی بینشی عمیق بیابد و از سختی و چالش‌های زندگی نهراسد. بنابراین هدف از پژوهش حاضر تعیین اثربخشی آموزش گروهی به روش تحلیل رفتار متقابل بر افزایش هوش معنوی زوجین شهر اصفهان بود. مواد و روش‌ها: پژوهش حاضر یک مطالعه نیمه تجربی از نوع پیش آزمون-پس آزمون همراه با گروه کنترل بود که در بین زوجین ارجاع داده شده به شوراهای حل اختلاف شهر اصفهان انجام گردید. نمونه آماری این پژوهش شامل 32 زوج بود که به روش نمونه‌گیری غیراحتمالی در دسترس انتخاب شده و به صورت گمارش تصادفی در گروه‌های آزمایش و کنترل گمارده شدند. سپس گروه آزمایش مداخلات آموزشی مربوط به تحلیل رفتار متقابل را در طی 10 جلسه آموزشی دریافت نمودند، در حالی که گروه کنترل از دریافت این مداخله در طول فرآیند پژوهش بی‌بهره بود. ابزار مورد استفاده در این پژوهش شامل پرسشنامه هوش معنوی کینگ (2008) بود. داده‌های جمع آوری شده بوسیله تحلیل کواریانس توسط بسته نرم افزاری SPSS20 مورد تجزیه و تحلیل قرار گرفت. یافته‌ها: نتایج تحلیل چند متغیره نشان داد آموزش تحلیل روابط متقابل توانسته منجر به افزایش معنی‌دار مولفه‌های تفکر وجودی انتقادی و تولید معنای شخصی گروه آزمایش در مقایسه با گروه کنترل در مرحله پس آزمون شود (0.001>P). نتیجه‌گیری: طبق یافته‌های این مطالعه می‌توان گفت مشاوره گروهی به شیوه تحلیل ارتباط متقابل، روش مناسبی برای افزایش هوش معنوی زوجین و در نهایت افزایش رضایتمندی آنان بوده که می‌توان با آموزش آن به ارتقاء کیفیت معنویت زوجین پرداخت.

Title: رابطه سواد سلامت با وضعیت سلامت عمومی، رفتارهای پیشگیرانه و استفاده از خدمات سلامت در منطقه بلوچستان

http://jech.umsha.ac.ir/article-1-122-fa.html

سابقه و هدف: سواد سلامتی عبارت است از مهارت هایی شناختی، اجتماعی که تعیین کننده انگیزه و قابلیت افراد در دستیابی، درک و به کارگیری اطلاعات به طریقی که منجر به حفظ و ارتقاء سلامت آن ها گردد. افراد با سواد سلامت ناکافی، وضعیت سلامتی ضعیف تری دارند. از این رو هدف این مطالعه، تعیین وضعیت سواد سلامت و ارتباط آن با وضعیت سلامت عمومی، استفاده از خدمات درمانی و اتخاذ رفتارهای پیشگیرانه بود. مواد و روش ها: این مطالعه توصیفی- تحلیلی بر روی 400 نفر از جمعیت 18 تا 65 سال شهر های بلوچستان انجام گردید. نمونه گیری بصورت خوشه ای چندمرحله ای انجام شد. داده ها با استفاده از ابزار سنجش سواد سلامت عمومی گردآوری شد. اطلاعات جمع آوری شده از طریق نرم افزار SPSS18  و با بکارگیری آزمون های آماری من ویتنی و کروسکال والیس مورد تجزیه و تحلیل قرار گرفتند. یافته ها: تنها 32 درصد شرکت کنندگان از سواد سلامت کافی برخوردار بودند. سطح سواد سلامت ناکافی در افراد با سنین بالا، میزان تحصیلات کمتر، درآمد پایین تر و زنان شایع بود. در این مطالعه، ارتباط آماری معنی‌داری بین سطح سواد سلامت و مراجعه به پزشک، انجام رفتارهای پیشگیرانه و وضعیت سلامت عمومی دیده شد. افراد با سطح سواد سلامت بهتر، وضعیت سلامت عمومی خود را بهتر ارزیابی کردند، همچنین بیش از سایرین نسبت به انجام رفتارهای پیشگیرانه مبادرت ورزیدند. نتیجه گیری: با توجه به پایین بودن سطح سواد سلامت گروه هدف، مداخلات آموزشی به منظور توسعه مهارت های سواد سلامت در جامعه ضروریست. تا بدین طریق بتوان تاثیرات منفی سطح سواد سلامت پایین را  در جامعه کاهش داد.

Title: تأثیر مشاوره بر رفتارهای پیشگیری کننده از پوکی استخوان در زنان مراجعه کننده به مراکز بهداشتی درمانی شهر همدان

http://jech.umsha.ac.ir/article-1-124-fa.html

سابقه و هدف: استئوپروز شایع ترین بیماری متابولیک استخوان است که با ایجاد اختلال ساختاری در استخوان، زمینه را برای شکستگی استخوان مستعد می سازد. این مطالعه به منظور بررسی تاثیر مشاوره بر اساس مدل اعتقاد بهداشتی بر رفتارهای پیشگیری کننده از پوکی استخوان در زنان مراجعه کننده به مراکز بهداشتی درمانی شهر همدان در سال­1394 انجام گردید. مواد و روش ها: مطالعه حاضر نیمه تجربی از نوع پیش آزمون و پس آزمون همراه با گروه کنترل بود که بر روی 80 زن مراجعه کننده به مراکز بهداشتی درمانی که به صورت تصادفی به دو گروه 40 نفره آزمون و کنترل تقسیم شده بودند، انجام گرفت. داده ها از طریق پرسشنامه استاندارد FFQ و دو پرسشنامه خودساخته دیگر جمع آوری و در سه مرحله (قبل، بلافاصله بعد و دو ماه بعد از مداخله) مورد ارزیابی قرار گرفتند. داده ها با استفاده از نرم افزار SPSS نسخه 18 و روش­های آماری توصیفی و استنباطی تحلیل شدند. یافته ها: بین دو­گروه قبل از مطالعه تفاوت معنی داری از نظر سن، سن منارک، تحصیلات، شغل و BMI وجود نداشت. قبل از مداخله تفاوت معنی داری بین میانگین نمرات سازه های مختلف این مدل در دو گروه مشاهده نشد. اما بعد از مداخله در گروه آزمون، میانگین نمره آگاهی و سازه های مختلف مدل به طور معنی داری افزایش پیدا کرد. نتیجه گیری: مدل اعتقاد بهداشتی می تواند در بالا بردن آگاهی، ایجاد حساسیت و درک شدت از پیامدهای یک بیماری یا یک رفتار غیر بهداشتی و درک منافع و موانع انجام یک رفتار صحیح و بهداشتی و انجام آن رفتار، مفید باشد.
